# Resveratrol improves osteogenic differentiation of senescent bone mesenchymal stem cells through inhibiting endogenous reactive oxygen species production *via* AMPK activation

**DOI:** 10.1080/13510002.2019.1658376

**Published:** 2019-08-23

**Authors:** Ting Zhou, Yurong Yan, Chenchen Zhao, Yao Xu, Qiong Wang, Na Xu

**Affiliations:** Institute of Biology and Medicine, College of Life Sciences and Health, Wuhan University of Science and Technology, Wuhan, People’s Republic of China

**Keywords:** Resveratrol, osteogenesis, bone mesenchymal stem cells, AMP-activated protein kinase, reactive oxygen species, osteoporosis, anti-aging, osteogenic differentiation

## Abstract

**Objective:** Resveratrol has been confirmed to improve bone quality and delay osteoporosis, but the mechanisms have not been thoroughly elucidated. In this report, we investigated the osteogenic differentiation effect of resveratrol on senescent bone mesenchymal stem cells (BMSCs) and the involvement of AMP-activated protein kinase (AMPK)/ reactive oxygen species (ROS) signaling pathway.

**Methods:** Cell senescence, viability, and osteogenic differentiation of BMSCs influenced by resveratrol were investigated and ROS production and AMPK expression were detected.

**Results:** Cell senescence, characterized by senescence β-galactosidase staining and senescence-related genes (p16, p21, and p53) expression, was attenuated by resveratrol. Cell viability, extracellular matrix calcification, and osteogenic-related genes expression were significantly enhanced after resveratrol treatment. ROS production in BMSCs was inhibited while AMPK expression was up-regulated by resveratrol. Inhibition of AMPK expression by compound C reduced resveratrol-prompted osteogenesis and ROS production down-regulation.

**Conclusion:** These results provide a potential mechanism involving AMPK activation/ROS inhibition signaling pathway in osteogenic differentiation of BMSCs enhanced by resveratrol. It suggests that development of therapy towards ROS is an effective way for osteoporosis treatment.

## 1. Introduction

Osteoporosis, an age-related degenerative disease characterized by the loss of bone mass and the structural deterioration of bone tissue, has become a social problem along with the exacerbation of global population aging [1]. The imbalance between the bone formation activity of osteoblasts and the bone resorption activity of osteoclasts is considered as an important reason for bone loss [2]. Bone mesenchymal stem cells (BMSCs) are considered the progenitors for osteoblasts, but, BMSCs will undergo a replicative senescence ‘Hayflick limit’ with generations of cell division [3]. The biological functions of residual MSCs progressively decline and become susceptible to the accumulation of cellular damage and senescence. The senescent BMSCs exhibit lower osteogenic differentiation and higher adipogenic differentiation [4,5]. Thus, it is an effective way for improving the bone quality of elderly people by enhancing the osteogenic differentiation activity of BMSCs.

Reactive oxygen species (ROS) are byproducts of biological reactions of energy generation, and are mainly produced in the mitochondria through the oxidative metabolism [6]. ROS is a double-edged sword for cells. Low level ROS, serving as intracellular signaling molecules, is necessary to maintain cell proliferation, differentiation, and self-renewability [7]. Conversely, excess ROS is harmful because of its potent ability to interact with a wide range of cellular molecules including DNA, RNA, proteins, and lipids [8]. Previous studies have shown that senescent cells have higher levels of ROS than normal cells, which implicated that ROS is involved in cell senescence [9]. The oxidative stress resulting from excessive ROS production is demonstrated to suppress the osteogenic lineage and increase the adipogenic terminal differentiation of BMSCs [10,11]. AMP-activated protein kinase (AMPK) is a fuel-sensing enzyme in response to oxidative stress to promote metabolic reprograming [12]. Several signaling pathways, including ERK, mTOR, Wnt/β-catenin signaling pathway, and energy metabolism, have been demonstrated involving in downstream regulation of AMPK-mediated BMSCs differentiation [13–15]. There is evidence for the role of AMPK in BMSCs differentiation by regulating ROS-related signaling cascades [14]. The up-regulation of AMPKα1 inhibits ROS production leading to enhanced osteogenic differentiation of Human adipose tissue-derived MSCs [16].

Recently, resveratrol (3,4,5-trihydroxystilbene), an edible polyphenolic phytoalexin, has attracted much attention in the anti-oxidation and anti-aging field because of its reductive phenolic hydroxyl group. Previous studies have shown that resveratrol stimulates the proliferation and differentiation of MC3T3-E1 cells in vitro, inhibited bone loss in ovariectomized rats in vivo, and promoted differentiation of normal BMSCs [17–19]. However, the mechanism is still unknown clearly. It is reported that resveratrol activates AMPK signaling pathway, but the function is cell-type dependent [20]. Therefore, we hypothesized that resveratrol might promote osteogenic differentiation of senescent BMSCs *via* AMPK/ROS signaling pathway. This research would help to find a way to rejuvenate the decreased osteogenic potential in aged BMSCs for enhancing bone quality.

## 2. Materials and methods

### 2.1. Cell separation and culture

Bone mesenchymal stem cells (BMSCs) were harvested from old (12 months) Kunming mice as follows. Femoral bones were collected under aseptic condition with the following cut off of bone terminals. The inner bone marrows were flushed out by α-MEM (Hyclone, GE Healthcare Life sciences, USA) with 10% fetal bovine serum (FBS, Gemini Bio-Products, Mexico). The separated cells were incubated in the culture medium at 37°C with 5% CO_2_ for 3 days. Then, the culture medium was changed to remove suspended cells. The adherent cells were cultured for 7 days to obtain the first passage cells. Cells were digested with 0.25% trypsin and passaged every 3 days. Cells within 3–5 passages were used in the following studies.

### 2.2. Cell viability assay

BMSCs were seeded into 96-well plate with the density of 5000 cells/well and treated with resveratrol (Sigma-Aldrich, USA, dissolved in DMSO) at different terminal concentrations (5, 10, 15, 20, and 25 μg/mL) for 48 h. Untreated cells were used as negative control (NC). Cells were stained with CCK8 kit (Beyotime Biotechnology, China) and the absorbance at 450 nm was measured by a microplate reader (Molecular Devices, SpectraMaxi3, USA).

### 2.3. Senescence β-galactosidase staining

The β-galactosidase was stained to evaluate the cell senescence level before and after the resveratrol treatment. BMSCs were cultured in 24-well plates for 12 h and exposed in different concentrations of resveratrol for 48 h. Cells were stained with Senescence β-Galactosidase Staining Kit (SA-β-Gal, Beyotime Biotechnology, China) according to the manufacturer’s instructions. The stained cells were imaged at the light field with an inverted fluorescent microscopy (DU80, Olympus, Japan). The aging proportion of BMSCs was calculated by counting the numbers of stained cells.

### 2.4. Detection of ROS production

BMSCs were seeded into 24-well plates and treated with different concentrations of resveratrol (5, 10, 15, 20, and 25 μg/mL) for 48 h. Then, cells were incubated with ROS assay kit (Beyotime Biotechnology, China) and the fluorescent images were captured with the inverted fluorescent microscopy. The fluorescence intensity was detected by the microplate system.

### 2.5. Extracellular matrix calcification detection

BMSCs were seeded into 24-well plates and cultured with osteogenic differentiation medium (Cyagen Biosciences, USA) after resveratrol treatment. After 3 days or 7 days cell culture, cells were fixed with 4% formaldehyde solution (Boster Biological Technology co. ltd, China) for 30 min and washed twice with PBS. Then cells were stained with alizarin red dye (Cyagen Biosciences, USA) for 5 min, washed twice with PBS, and imaged in the light field to observe the calcium nodules. To quantitatively analyze the calcification, 2 mL cetylpyridinium chloride (Sigma-Aldrich, USA) was added into each well to dissolve the calcium nodules. The absorbance at 562 nm was measured.

### 2.6. Real-time quantitative polymerase chain reaction (qRT-PCR) detection

The mRNA expression of senescence-related genes (p16, p21, and p53), osteogenic-related genes (ALP, Col-I, OCN, OPN, and Runx2), and signaling pathway-related genes (AMPKα1 and AMPKα2) in BMSCs were detected by qRT-PCR. BMSCs were seeded in 6-well plates and cultured with the culture medium containing different concentrations of resveratrol for 48 h to detect senescence-related genes. BMSCs were seeded in 6-well plates and cultured with osteogenic culture medium containing different concentrations of resveratrol for 7 days to detect osteogenic-related genes and signaling pathway-related genes. The qRT-PCR process was performed as follows. The total mRNA was extracted by TRIzol (Aidlab Biotechnologies Ltd. Co., China), and then reverse-transcribed to cDNA using a reverse transcription kit (Takara Biomedical Technology Ltd. Co., China). The DNA was probed by SYBR Green fluorescent dye (Takara, China). The primers were designed as shown in Table 1. The PCR reaction program was pre-denaturation at 95°C for 3 min, with following denaturation at 95°C for 10 s, refolding at 55°C for 30 s for 40 cycles, and extension at 72°C for 30 s. The system was detected by a real-time fluorescence quantitative PCR instrument (Bio-Rad, USA). Housekeeping gene GAPDH was used as control.

**Table 1.** Primers for qRT-PCR.

Genes	Forward Primers (5′-3′)	Reverse Primers (5′-3′)
p16	GCGCTCTGGCTTTCGTG	CACTACCTTCTCCCGCCC
p21	TGTTCCGCACAGGAGCAAAG	CGAAGTCAAAGTTCCACCGT
p53	CCCAGGATGTTGAGGAGTTT	TGAGAAGGGACAAAAGATGA
ALP	TGACATCCCAGAAAGACACC	CGTTCACCGTCCACCACC
Col-I	ACGCCATCAAGGTCTACTG	ATCCATCGGTCATGCTCT
OCN	GAGGGCAATAAGGTAGTGAA	CATAGATGCGTTTGTAGGC
OPN	TTTCACTCCAATCGTCCC	TTAGACTCACCGCTCTTCAT
Runx2	TACTTCGTCAGCATCCTATCA	TTCCGTCAGCGTCAACAC
AMPKα1	CCCCTATTATTTGCGTGTA	CTGTGGAGTAGCAGTCCCT
AMPKα2	ATCTCAACCGTTCTGTCGC	GGGGTCTTCAGGAAATAGG
GAPDH	GTGTTTCCTCGTCCCGTAG	AAAGTGGAGATTGTTGCCAT

### 2.7. Evaluation of AMPK/ROS signaling pathway on osteogenic differentiation of BMSCs

To study the effect of resveratrol on AMPK/ROS signaling pathway, the protein expression levels of AMPK and p-AMPK were detected with western blot experiments. BMSCs were cultured with/without 15 μg/mL resveratrol and then 5 μM Compound C (CC, Selleck, USA), an AMPK inhibitor, were added into the BMSCs culture for 48 h. Total protein is extracted after cells were lysed by RIPA (with 1% protease inhibitor PMSF) and the total protein concentration was determined by BCA kit (Beyotime Biotechnology, China). After SDS-PAGE electrophoresis, proteins were incubated with antibodies and then imaged with chemiluminescence imaging system (Bio-Rad, USA).

Simultaneously, ROS production and extracellular matrix calcification were also detected to verify AMPK signaling pathway. BMSCs were exposed in 15 μg/mL resveratrol and 5 μM CC and cultured with osteogenic culture medium for 7 days. Then, alizarin red staining was performed to evaluate the calcification of BMSCs. BMSCs were exposed in 15 μg/mL resveratrol and 5 μM CC and cultured with the culture medium for 48 h. Then, ROS production was detected according to the above method.

### 2.8. Statistical analysis

All experiments were performed at least three times. Data were expressed as mean ± standard deviation (SD). ANOVAs were used for statistical analysis and *p* < 0.05 was identified as significant difference.

## 3. Results

### 3.1. Effect of resveratrol on cell senescence

**Figure 1.**
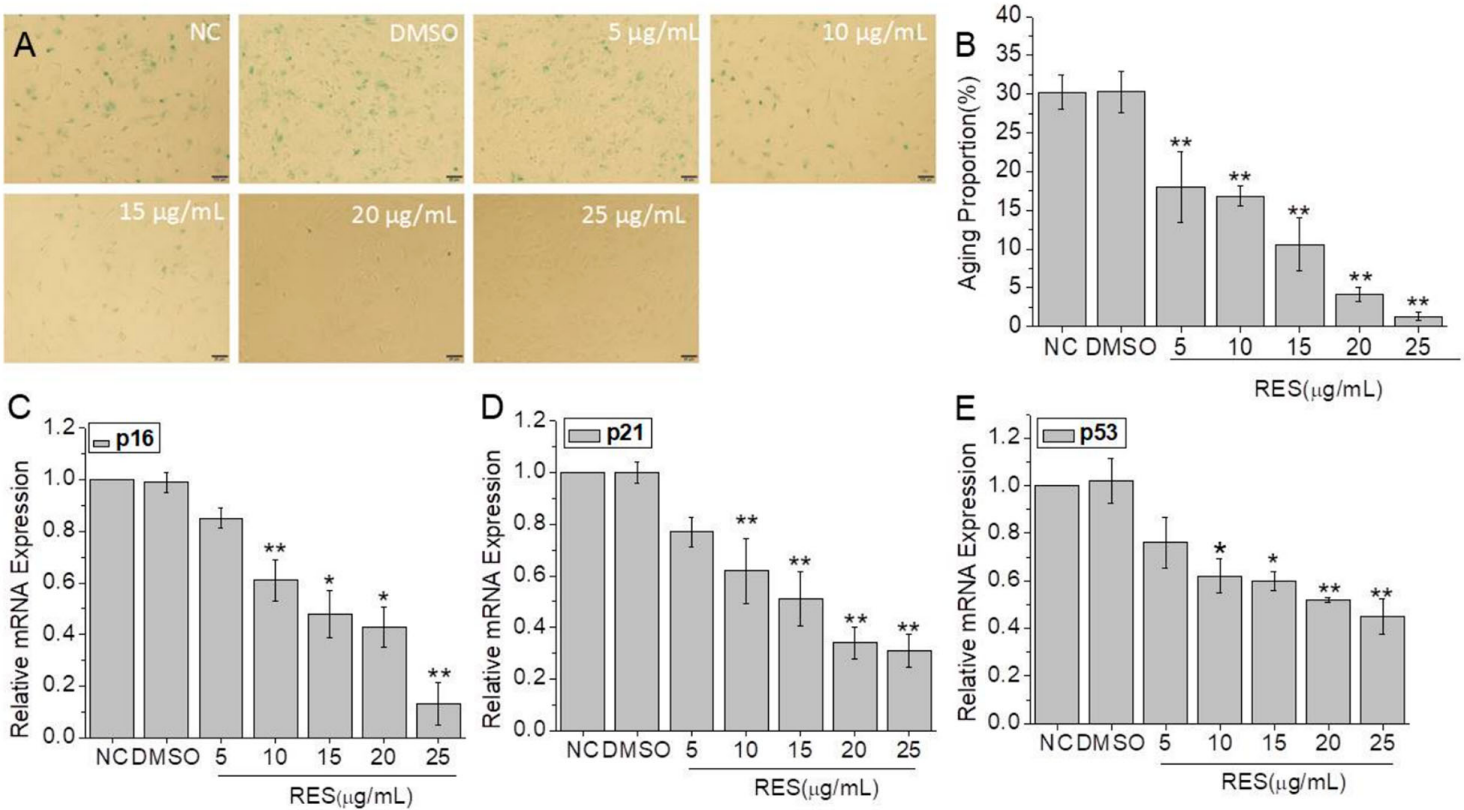
The anti-senescence effect of resveratrol on BMSCs after 48 h treatment at the concentration of 5, 10, 15, 20, and 25 µg/mL, respectively. (A) Senescence β-galactosidase staining of BMSCs (Scale bar: 100 µm). (B) Cell aging proportion. (C), (D), and (E) Relative mRNA expression of senescent genes p16, p21, and p53. GAPDH was used as control. (* and **means *p* < 0.05 and *p* < 0.01 compared to NC, respectively).

In order to study the effect of resveratrol on cell senescence, we tested the SA-β-Gal production (Figure 1(A,B) and the mRNA expression levels of senescence-related genes including p16, p21, and p53 (Figure 1(C–E). The SA-β-Gal staining results showed that the number of stained cells decreased with the increase of resveratrol concentration. The lowest senescent cell ratio was observed at 25 µg/mL resveratrol, showing a 96% decrease compared to NC group. Similarly, the mRNA expression levels of senescence-related genes were down-regulated with resveratrol treatment. Among them, p16 gene expression was 86% lower than NC group. These results proved that resveratrol inhibits BMSCs senescence in a concentration-dependent manner.

### 3.2. Effect of resveratrol on cell viability and ROS production

**Figure 2.**
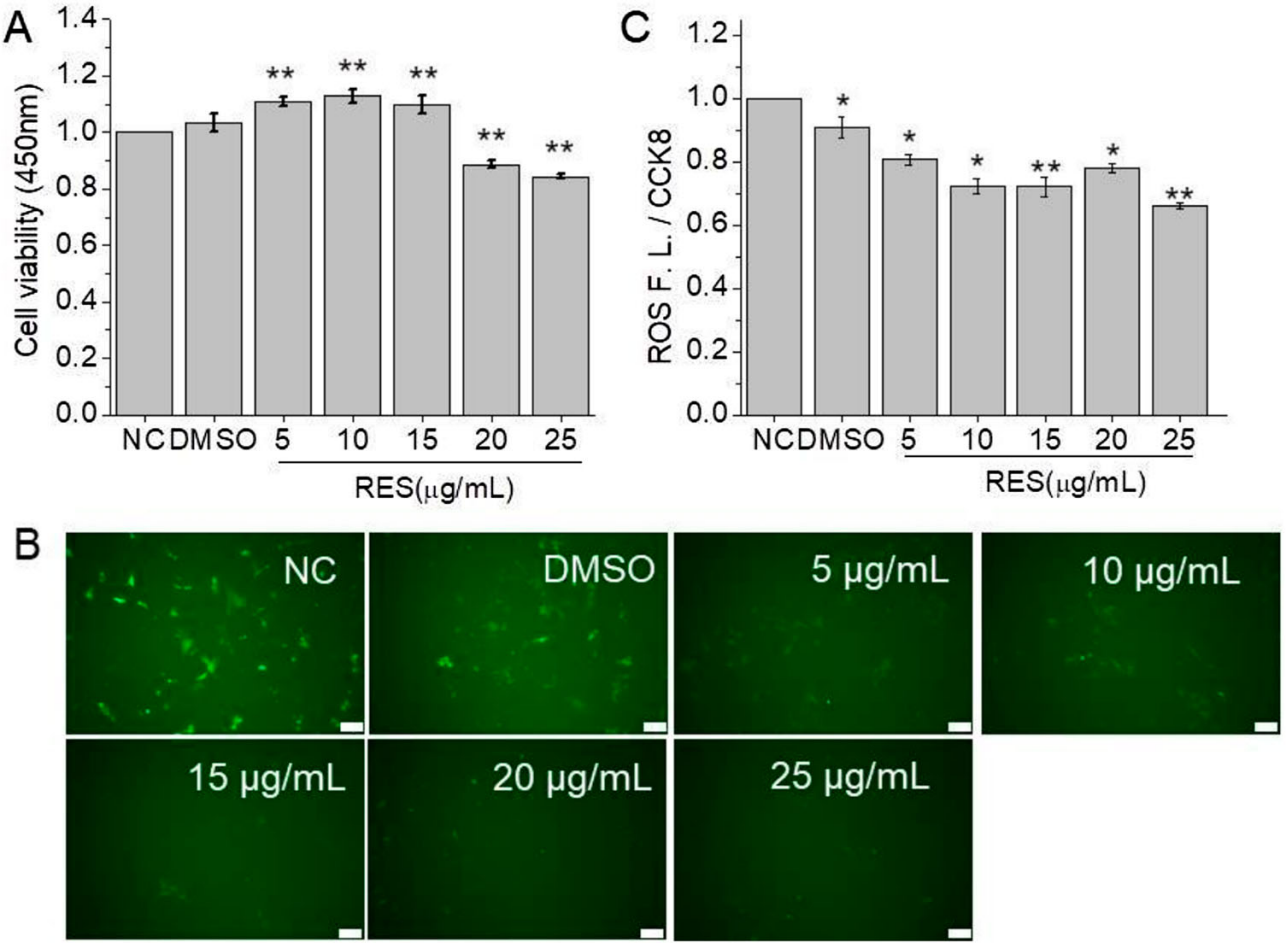
The effect of resveratrol on cell viability and ROS production after 48 h treatment at the concentration of 5, 10, 15, 20, and 25 µg/mL, respectively. (A) Cell viability detected by CCK8 assay. (B) Fluorescent images of BMSCs stained with ROS test kit (Scale bar: 100 µm). (C) Relative fluorescent intensity of ROS detected by a multifunctional microplate reader. (* means *p* < 0.05, ** means *p* < 0.01, compared to NC).

To estimate the effect of resveratrol on cell viability, CCK8 assay was performed. As shown in Figure 2(A), at 48 h post of resveratrol treatment, cell viability showed significant increase compared to NC group at 5, 10, and 15 µg/mL resveratrol while the higher concentration of resveratrol slightly inhibited BMSCs viability. The results suggested that resveratrol showed a bimodal effect on cell viability and the concentration lower than 15 µg/mL is beneficial for cell viability.

ROS production has been proved to be related with cell differentiation and senescence. Herein, we detected ROS production level of BMSCs after resveratrol treatment. The fluorescent images of ROS labeling (Figure 2(B)) showed that intracellular ROS production per cell was reduced with the increase of resveratrol concentration. The quantitative analysis for fluorescent intensity (Figure 2(C)) also proved the conclusion.

### 3.3. Resveratrol promotes osteogenic differentiation of BMSCs

**Figure 3.**
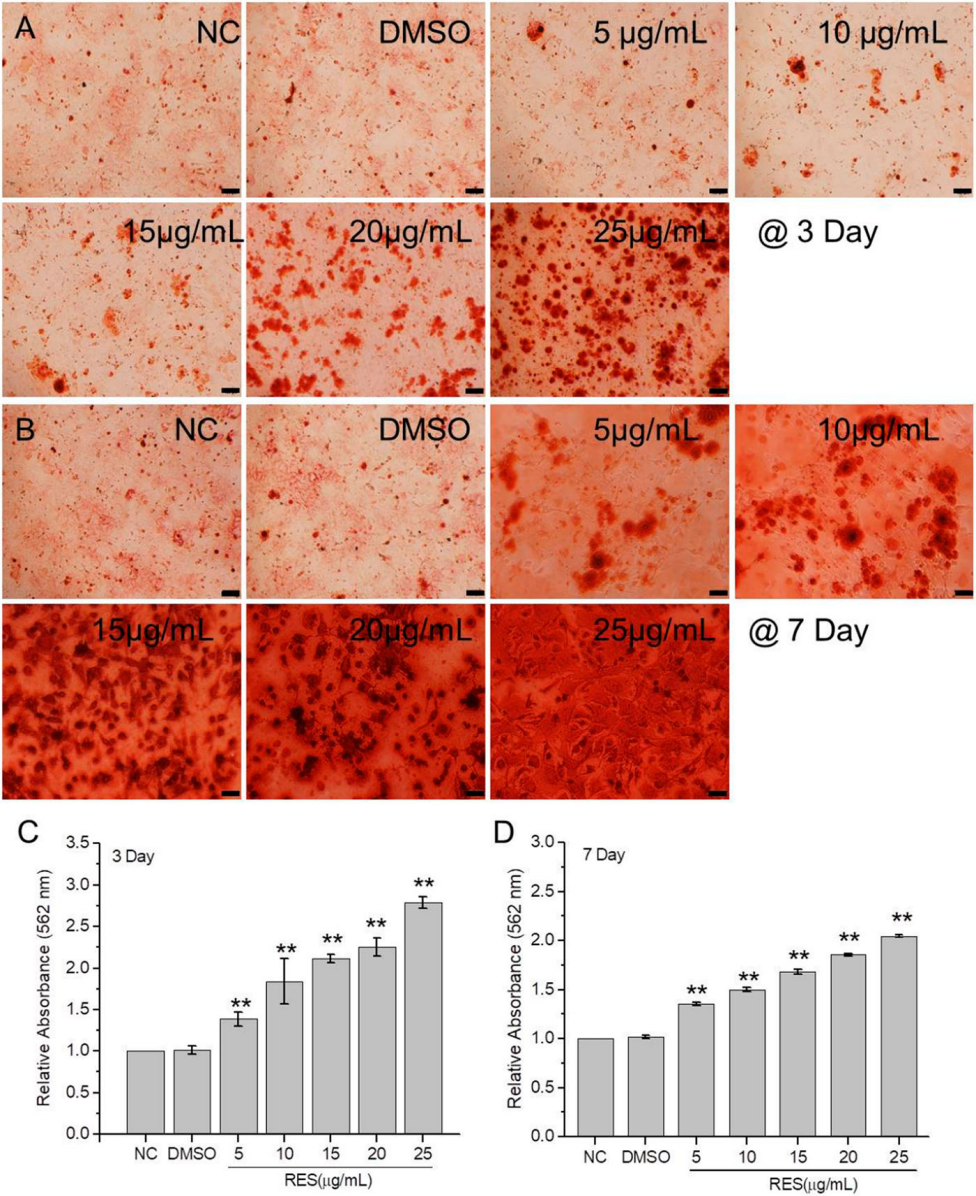
Extracellular matrix calcification of BMSCs after treated with resveratrol at the concentration of 5, 10, 15, 20, and 25 µg/mL, respectively. (A) and (B) BMSCs stained with alizarin red after 3 and 7 days treatment. (C) and (D) Relative OD values at 562 nm of calcium nodules dissolved with cetylpyridinium chloride after 3 and 7 days treatment, respectively. (Scale bar: 100 µm) (* means *p* < 0.05, ** means *p* < 0.01).

The extracellular matrix calcification level and osteogenic-related genes expression were detected to estimate BMSCs osteogenic differentiation. The alizarin red staining results (Figure 3) showed that calcium nodule number obviously increased with resveratrol concentration at 3 and 7 days culture. The relative quantity of calcium nodules was detected after digestion with cetylpyridinium chloride. The maximum increment of 2.79 times was observed at 3 days culture with 25 μg/mL resveratrol compared to the control. The increment at 7 days culture with 25 μg/mL resveratrol was 2.09 times, which is a little lower than 3 days culture.

**Figure 4.**
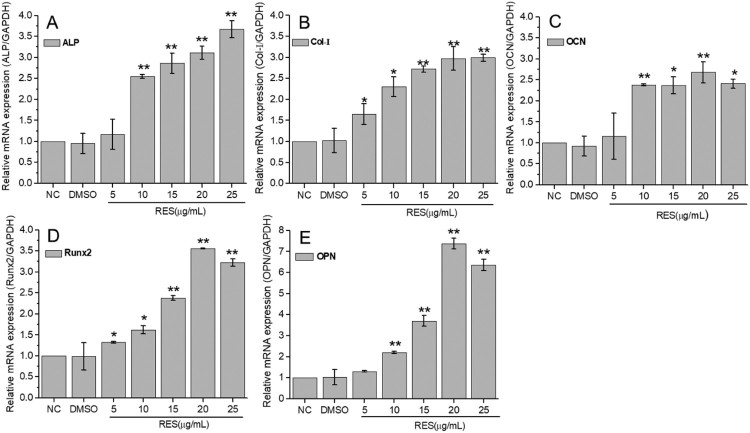
Osteogenic-related gene expression of BMSCs detected by qRT-PCR after 7 days treatment with resveratrol at the concentration of 5, 10, 15, 20, and 25 µg/mL, respectively. (A) ALP, (B) Col-I, (C) OCN, (D) Runx2, (E) OPN. GAPDH was used as internal control. The results were expressed as fold changes in mRNA abundance compared to NC. (* means *p* < 0.05, ** means *p* < 0.01).

The qRT-PCR results at 7 days culture were displayed in Figure 4. The mRNA expression levels of ALP, OCN, and OPN were significantly up-regulated at resveratrol concentration of higher than 10 μg/mL, while Col-I and Runx2 were significantly up-regulated at resveratrol concentration of higher than 5 μg/mL. The expressions of selected osteogenic-related genes increased with resveratrol concentration. Most maximum increments of 2.68∼3.67 times appeared at 20 μg/mL or 25 μg/mL resveratrol, especially 7.37 times for OPN at 20 μg/mL resveratrol. These results demonstrated that resveratrol promotes osteogenic differentiation of BMSCs.

### 3.4. Resveratrol activates AMPK/ROS signaling pathway on osteogenic differentiation of BMSCs

**Figure 5.**
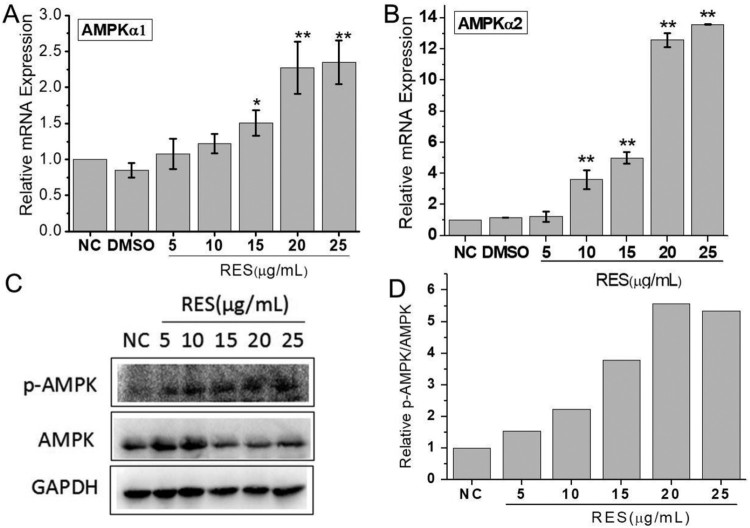
Resveratrol activates AMPK pathway. (A) and (B) were mRNA expressions of AMPKα1 and AMPKα1 through qRT-PCR detection after BMSCs were treated with different concentrations of resveratrol. GAPDH was served as the internal reference. (C) Western blot analysis of p-AMPK and AMPK protein expression. (D) The quantitative analysis of western blot results.

ROS production has been found to be negatively regulated by AMPK pathway, so we chose an AMPK inhibitor and detected AMPK expression. AMPKα1 and AMPKα2 gene expression were both significantly up-regulated when cells were treated with 15 μg/mL resveratrol and the increase level was dependent on the resveratrol concentration (Figure 5(A,B). The western blot analysis exhibited that the phosphorylated AMPK (p-AMPK) expression was significantly increased with the resveratrol concentration (Figure 5(C)). The quantitative analysis showed the relative p-AMPK/AMPK increased 5.5 times at 20 μg/mL resveratrol compared to NC.

**Figure 6.**
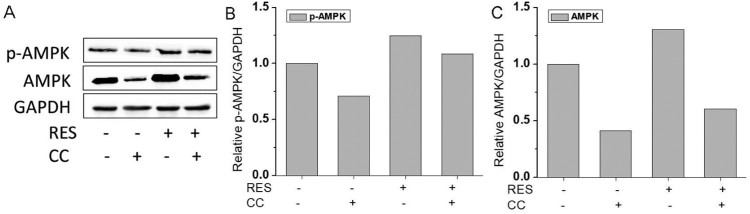
AMPK expression in BMSCs after 48 h treatment with 15 µg/mL resveratrol and/or compound C (CC). (A) Western blot image of AMPK protein expression after treatment with resveratrol and/or CC. (B) and (C) were quantitative analysis of western blot results.

**Figure 7.**
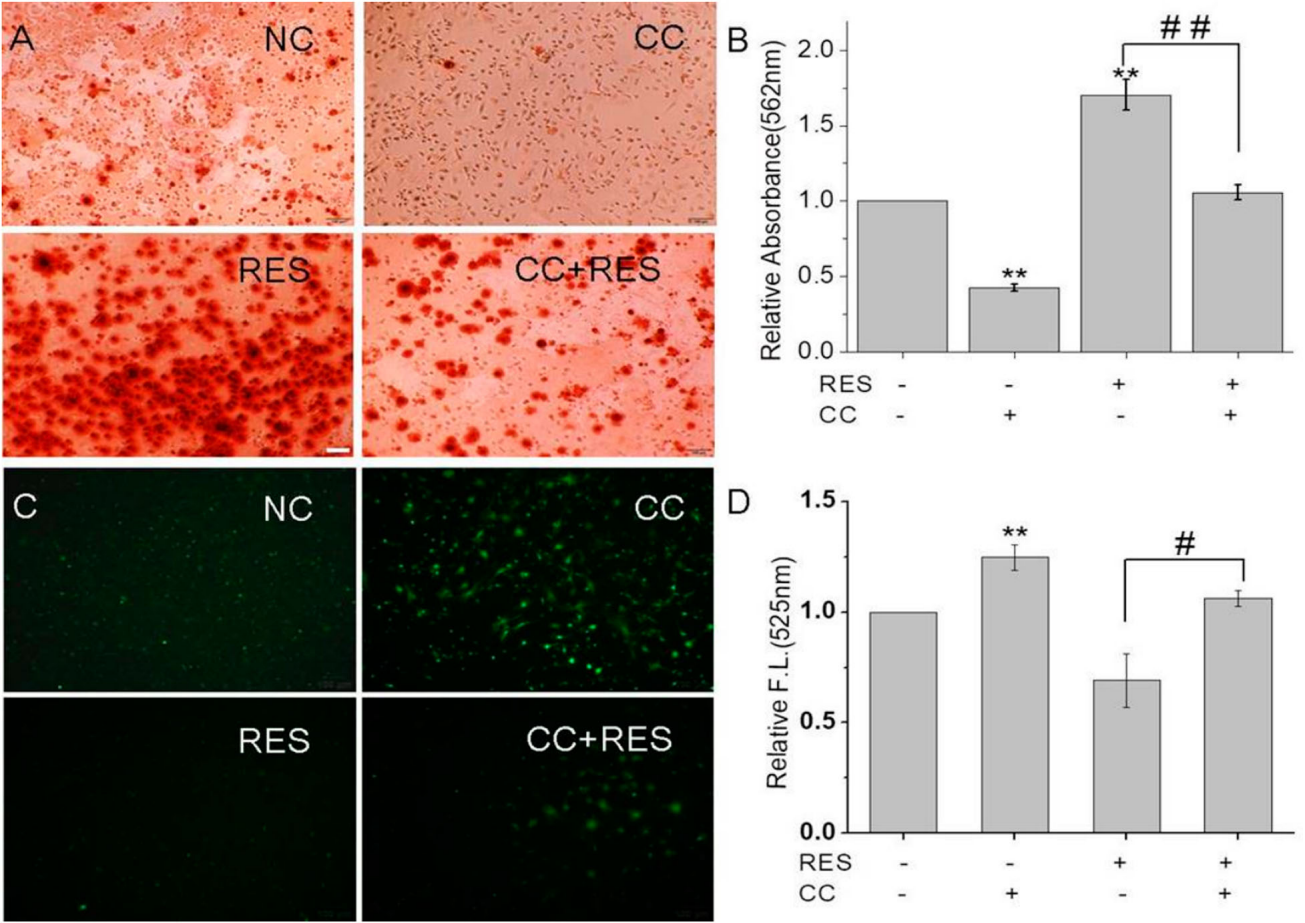
Calcification and ROS production in BMSCs after 48 h treatment with 15 µg/mL resveratrol and/or compound C (CC). (A) and (B) were alizarin red staining images and quantitative analysis of calcification. (C) and (D) were ROS production fluorescent images and quantitative analysis of fluorescence intensity. * means *p* < 0.05 compared to RES-/ CC-, ** means *p* < 0.01 compared to RES-/CC-, # means *p* < 0.05 compared to RES+/CC-, ## means *p* < 0.01 compared to RES+/CC-. (Scale bar: 100 µm).

We further investigated the effect of AMPK/ROS signaling pathway on the osteogenic differentiation of BMSCs by using an AMPK inhibitor, CC. The results (Figure 6) showed that the protein expression levels of AMPK and p-AMPK showed the following tendency: RES+/CC- > RES-/CC- > RES+/CC+ > RES-/CC+. The alizarin red staining for BMSCs after CC treatment (Figure 7(A,B)) displayed that CC decreased resveratrol-prompted extracellular matrix calcification of BMSCs. Simultaneously, the inhibition of resveratrol-induced ROS production was reduced after inhibiting AMPK expression (Figure 7(C,D)). These results demonstrated that resveratrol promotes the osteogenic differentiation of BMSCs through AMPK/ROS signaling pathway.

## 4. Discussion

Resveratrol has attracted much attention due to its multiple functions such as anti-oxidation, anti-inflammation, anti-cancer, and anti-aging. It also has been described to be related with bone formation. Resveratrol inhibits osteoclast activity, simulates osteoblast differentiation, and protects against bone loss in ovarectomized rats [17–19]. In our studies, resveratrol showed a dose-dependent and bimodal effect on cell viability. When the concentration is lower than 15 µg/mL, cell viability was increased and when the concentration is higher than 20 µg/mL, cell viability was decreased. The result is consistent with previous study of promoting osteogenesis of human mesenchymal stem cells, in which the resveratrol concentrations are chosen lower than 20 µg/mL [17]. The cell senescence characterized by SA-β-Gal staining and related gene p16, p21, and p53 were significantly down-regulated with resveratrol treatment in the range of 5 to 25 µg/mL. Furthermore, the extracellular matrix calcification level and osteogenic-related genes expression were significantly improved after resveratrol treatment and the highest improvement appeared at 20 or 25 µg/mL. These results give a direct proof of resveratrol on enhanced osteogenic differentiation of senescent BMSCs.

Many studies have pointed out the importance of ROS on directing BMSCs differentiation [11,21,22]. ROS is a kind of metabolic side products with unpaired electrons, including hydroxyl radical (OH^-^) and superoxide anion (O_2_^-^). So ROS is unstable and capable of initiating oxidation, causing various cellular responses. For BMSCs, moderate ROS level is vital for maintaining cell self-renewal and proliferation, while excessive ROS leads to apoptosis or senescence with loss of BMSCs function [23]. It is known that senescent cells show higher levels of ROS than normal cells [9], so we investigated the antioxidation effect of resveratrol on senescent BMSCs. The results showed that the endogenous ROS in senescent BMSCs significantly decreased while calcified extracellular matrix increased at 25 μg/mL resveratrol compared to NC group. It is suggested that resveratrol improves osteogenesis *via* inhibiting ROS production.

The molecular mechanism was further explored in this report. AMPK is an energy sensor and plays important role in metabolic homeostasis. It has been proved that resveratrol activates AMPK *via* the activation of SIRT1, a catalyzer for NAD^+^-dependent protein deacetylation [14,24,25]. A recently research further found that resveratrol-induced AMPK activation was not be solely through activation of SIRT1, but also through an integrated effect of SIRT1-liver kinase B1 (LKB1)-AMPK [20]. In addition, the activation mechanisms vary among cell types and in some cell types, resveratrol fails to activate AMPK [20]. Our results showed that the expression levels of AMPKα1, AMPKα2 and p-AMPK/AMPK were all significantly increased with the treatment of resveratrol, which suggested that resveratrol could activate AMPK in BMSCs. However, a previous study found that AMPKα2 was not consistently detected and increased during BMSCs osteogenesis [26]. This discrepancy may be related to different cell types and different drug treatment methods. Human adipose tissue-derived mesenchymal stem cells and rat bone mesenchymal stem cells are derived from different tissues and it is very likely that the transcription profiles are quite different between them. On the other hand, resveratrol-induced osteogenesis may also adopt different pathway with naturally aging cells.

**Figure 8.**
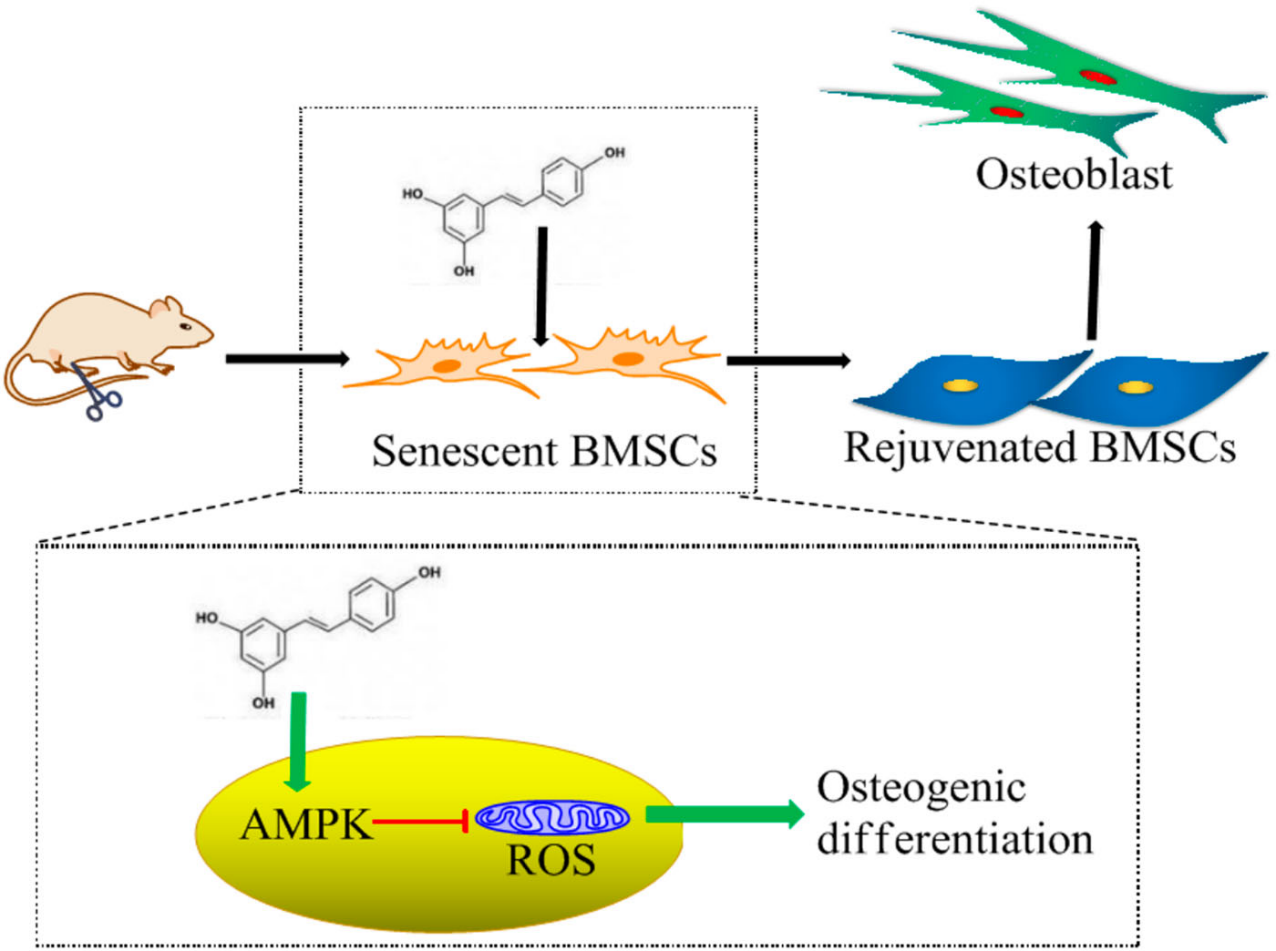
Molecular mechanism of resveratrol-promoted osteogenesis in aging rats. Resveratrol facilitates osteogenic differentiation of aging BMSCs through attenuating cell senescence and down-regulating ROS production via AMPK activation.

It has been found that AMPK mediates metabolic reprograming and redox state [12]. We used an AMPK inhibitor CC to investigate the relationship between AMPK expression and ROS production. The results showed that when AMPK expression was inhibited by CC, resveratrol-induced inhibition on ROS production in BMSCs were significantly decreased as well as the promotion on the osteogenic differentiation. There are many downstream effectors responsible for AMPK-mediated ROS inhibition, including FOXO, NADPH, and mTOR. AMPK facilitates FOXO phosphorylation and acetylation, enhancing its translocation to nucleus and the transcriptional activity of antioxidant genes [27,28]. FOXO activation increases ability against oxidative stress by targeting the expression of antioxidant enzymes, such as SOD, catalase and sestrin [29]. AMPK promotes NADPH production to increase the recyclability of GSH, an abundant antioxidant in cells. In addition, AMPK inhibits mTOR, an important signaling pathway relating with cell senescence [30]. From the above, it is concluded that resveratrol facilitates osteogenic differentiation of senescent BMSCs and this function depends on AMPK-mediated inhibition of ROS production and attenuation of cell senescence (Figure 8).

## 5. Conclusion

In this report, we found that resveratrol attenuated BMSCs senescence derived from aging rats and leads BMSCs differentiation toward osteoblast lineage. AMPK activation-induced down-regulation of ROS production was responsible for this phenomenon. This study helps to demonstrate the molecular mechanism involved in resveratrol-regulated BMSCs fate and may provide new insight into the drug development towards ROS/age-induced osteoporosis.
